# Supplementary material for: Integration of small RNA, degradome and proteome sequencing in Oryza sativa reveals a delayed senescence network in tetraploid rice seed
Source: PLoS One. 2020 Nov 13;15(11):e0242260. doi: 10.1371/journal.pone.0242260 (PMC7665819; doi:10.1371/journal.pone.0242260)
Supplement: Attachment — Submitted filename: Response to Reviewers (PONE-D-20-22960).doc [file pone.0242260.s004.doc]

Dear editor,

We received the reviews from two reviewers on September 11, 2020. First of all, we would like to thank you and the two reviewers very much for giving us valuable suggestions about revising our paper (PONE-D-20-22960). We have earnestly revised this paper according to the comments made by you and the two reviewers. Here, the specific revisions are highlighted in our manuscript and the answers are shown in detail as follows:

Editor's comments:

Reviewer #1: The manuscript describes a technically sound scientific study with data to support the findings. Experiments were performed with appropriate controls, repetitions and sample sizes. Conclusions are duly drawn from the data presented. The statistical analysis been performed appropriately. The authors have made fully available all the data underlying the conclusions in their manuscript. The manuscript presented in an intelligible fashion and written in standard English.

Answer: Thank you very much for your reviews of our manuscript (PONE-D-20-22960). I'm very excited to get your approval on experiment design, statistical analysis, Writing, etc. This manuscript foucs on the delayed senescence network in tetraploid rice seed, which is one of our research sections. Our team has been working on rice for nearly 20 years, and we want to get a more clear understanding of rice through ongoing research.

Reviewer #2: This manuscript described a comprehensive comparison between tetraploid and diploid rice accessions via miRNA-seq together with degradome and proteome sequencing analyses. The authors provide preliminary data showing that miR164d and some other small RNAs may play roles in regulation of the activity of antioxidant enzymes, leading to determining of seed senescence and seed longevity. However, I did not found any degradome and proteome data to support the claims throughout the manuscript. Besides, I have the following concerns to argue the publishable of this manuscript.
Answer: Thank you for your good suggestion. In the manuscript, Fig.5 shows the changes of small RNAs in 9311-2x vs 9311-4x according to the degradome sequencing data, miR164d was involved in RNA degradation pathway. Table.3 shows the identified antioxidant enzyme proteins according to proteome data, and the expression of these proteins are relevant with antioxidant enzyme activities.

The following revisions are shown in detail as follows:

There are many confusing or unclear sentences, I listed some below.
Lines 42-64, the two sentences here are confusing.

Answer: Thank you for your good question. Because there are more than two sentences from lines 42 to 64, I guess you mean lines 42 to 44 . For these two sentences (This irreversible change is due to the process of seed senescence [2]. The use of artificial aging methods has become a common method to investigate the mechanisms of seed senescence, the influences of seed vigor, RNA integrity, DNA repair, etc. [3,4]), I have replaced the word "effect" with "change" and "understand" with "investigate", meanwhile a word "and" was deleted. Firstly, as the process of seed senescence, some irreversible changes will occur. Secondly, many scholars are using artificial aging methods to investigate the mechanisms of seed senescence, the influences of seed vigor, RNA integrity, DNA repair, etc. (Lines 42-44)
Lines 84-90, the long sentence here is confusing.

Answer: Thank you for your good question. I have changed the long sentence into three short sentences and restate as follows :

The control of low temperature, low humidity and good ventilation conditions can ensure the safe storage of grain crop seeds. In order to satisfy the above -mentioned conditions, we need to build some high standard seed storage facilities, which will consume a lot of energy or resources. Improving the seed storability may be a cost-effective strategy. (lines 84-87)
Lines 97-98, the mean of the sentence “With…decline” is unclear.

Answer: Thank you for your good question. I have revised this sentence, which I want to state clearly the changes related to RNA as follows:

With the destruction of RNA integrity, the function of RNA transport and signal transduction will be lost, and the ability of RNA to regulate protein synthesis will also decrease. (lines 94-96)
Line 121, “identifying” is not a correct word here, how can “mechanisms” be identified? I suggest to use “understanding” instead.

Answer: Thanks for your good suggestions. I have revised it according to your's comments and replaced “identifying” with “understanding”(line 120).
Lines 343-344 and Fig 6, the gene IDs for SOD, CAT and POD are not clear. I suggest that the authors use either RAP locus (for example, Os02g0672200) or MSU locus (LOC_Os02g45070), so that the readers can see what genes are been talking about.

Answer: Thanks for your good suggestions. I have revised the gene IDs with RAP locus .(lines 366-374)  
Lines 364-368 and Table 3, I cannot understand what the protein accession numbers are used. Are the numbers from the database of LC-MS/MS or from any public databases? If these numbers are from the database of LC-MS/MS, please change to numbers corresponding to those of a public database. If the numbers are from a public database, please point out what database is used.

Answer: Thank you for your good question. The protein accession numbers are from the public database of japonica_NCBI_1903（280289 sequences）

Other concerns:
Lines 53-54 and throughout the manuscript, delete the “-” between “miR” and the “number”. The name of miRNAs should follow the previous reports.

Answer: Thanks for your good suggestions. I have deleted the “-” between “miR” and the “number” in lines 53-54 and throughout the manuscript. (lines 53-54)
Lines 103-111, I suggest to move “MicroRNAs…process” into a new paragraph.

Answer: Thanks for your good suggestions. I have revised it according to your's comments and moved “MicroRNAs…process” into a new paragraph.( Lines 101-109)
Lines 110-111, the statement of the sentence “Previous studies…” seems not consistent with the context and the cited reference (33) is incorrect.

Answer: Thank you for your good question. This sentence is to state the understanding of my previous research (unpublished data). I found that the gene expression of 9311-2x and 9311-4x of rice seeds was different during the artificial aging treatment (0, 4, 8, 12, 16 days), and I speculate the protein expression of 9311-2x and 9311-4x was also different.The references cited here are incorrect. I have deleted the references here and updated the reference numbers of the full text. I deleted “and protein”, in order to express more rigorously.（Lines 108-109）

Lines 118-119, I cannot understand how proteomics can be used to verify the miRNA and degradome sequencing results. RNA sequencing data should be verified by RT-PCR.

Answer: Thank you for your good question. Joint analysis of different sequencing data is a common method for transcriptional regulation research，In this paper, mutual corroboration of the protemics,, the miRNA and the degradome sequencing results were used for further analysis a delayed denescence network in tetraploid rice seed. In order to offer a more accurate description, I have revised this sentence as follows:

The joint analysis of the proteomics, the miRNA and the degradome sequencing results may provide a more powerful explanation for a delayed denescence network in tetraploid rice seed.（lines 116-118）

Lines 70-74 and 125-128, the authors reasoned that PMeS rice lines have advantages, I am suspicious that the polyploidy 9311-4x is with high seed setting. I suggest that the authors show the panicle images to display this trait.

Answer: Thank you for your good question. In our previous study, a lot of PMeS lines have been used for the delayed senescence analysis of rice seed. The results showed that A3 (PMeS line with high seed setting) and 9311-4x had better tolerance (unpublished data). Although the tetraploid rice of 9311-4x is not a PMeS rice line, 9311-4x was choosed for the delayed senescence analysis rice seed together with the diploid rice of 9311-2x, considering the consistence of genetic background. After multiple generations of continuous selection, the rice seed setting rate of 9311-4x has reached about 50%, which can satisfy the experimental requirements in addition.

Line 136, “6000lx” should be “6000 lx”.

Answer: Thanks for your good suggestions. I have revised it according to your's comments.(Line 135)
Line 137, “20 seeds for” should be “Twenty seeds were used for”

Answer: Thanks for your good suggestions. I have revised it according to the reviewer's comments. (line 136)
Lines 221-223, antioxidant enzyme activity assays should cite references or give a brief description of the method.

Answer: Thank you for your good question. I've added the reference paper .This paper is as follows:

Zhiyou K, Yeju L, Peng Q . Effects of Accelerated Aging on CAT,POD,SOD Activities and Soluble Protein Content of Wheat Seeds[J]. Journal of the Chinese Cereals and Oils Association, 2010, 25(10):24-27.

I've added a brief description of the method as follows：

Extraction of enzyme solution: Take 0.1g rice seed, add 1ml 0.05mol/l phosphate buffer (pH=7.8) , then grind in ice bath and centrifugal treatment at 10000 rpm/min for 20 min, the supernatant should preserved at 4 ℃ for use.（lines 223-225）

Determination of superoxide dismutase (SOD) activity: Take 200μl enzyme solution, add 3ml 0.05mol/L phosphate buffer (pH 7.8), 600μl 130mmol/L methionine solution, 600μl 750μmol/L nitro-blue tetrazolium(NBT) solution, 600μl 100 μmol/L riboflavin solution, 600μl 100μmol/L EDTA-Na2 solution, and reacted for 20 min under 4000lx of light. The absorbance was measured at 560nm by spectrophotometer（lines 226-230）.

Determination of catalase (CAT) activity: Take 50 μl enzyme solution and 2ml 0.1mol/lphosphate buffer (pH=7.0) , 25 ℃ water bath for 5min, add 0.5ml 0.1mol/l H2O2, vortex mixed, The enzyme activity was measured at 240nm and read every 20s for 3 times. The absorbance change value per minute (ΔA240/min·g FW) was used to represent the enzyme activity. （lines 231-234）

Determination of peroxidase (POD) activity: Take 20 μl enzyme solution and 2ml 0.3% guaiacol, 30 ℃ water bath for 5min, 1ml of 0.3% H2O2 was added, and vortex mixed. The enzyme activity was measured at 470 nm and read every 20s for 3 times. The absorbance change value per minute (Δ A470/min·gFW) was used to represent the enzyme activity（lines 235-238）.

Line 291, here should cite previous published papers because of the claim “consistent with previous studies in O. sativa”.

Answer: Thanks for your good suggestions. I have added the previous published paper titled “Identification and Functional Analysis of Flowering Related microRNAs in Common Wild Rice (Oryza rufipogon Griff.)”. In this paper, sRNA sequences with lengths of 21 nt were found significantly more frequently than those with other lengths, accounting for 23.73%, 30.41% and 21.70% in the three datasets, respectively. This paper is as follows:

Zongxiang C , Fuli L , Songnan Y , Yibo D, Qianhua Y, Feng W, Weimin L, Ying J, Shirong J, XinWu P. Identification and Functional Analysis of Flowering Related microRNAs in Common Wild Rice (Oryza rufipogon Griff.)[J]. Plos One, 2013, 8(12):1-16. (line 306)

Lines 305-306 and table 1, I do not think that miR164d targets a gene encoding Cu-Zn superoxide. I suggest that the authors double check and cite a reference for this claim. I also suggest that the authors double check the fold change (FC) value in table 1 and clarify whether they are log2FC.

Answer: Thank you for your good question. The results of degradome sequencing analysis showed that MiR164d target four genes, including Os02g0579000, Os06g0675600, Os12g0610600, and Os04g0460600. These four genes are members of NAC transcription factor gene family. The interaction analysis shows that gene Os12g0610600 (OsJ_36832) interact with gene Os04g0573200 (CCS), copper chaperone for superoxide dismutase, chloroplastic-like [Oryza sativa Japonica Group (Japanese rice)]. Further analysis shows that gene Os04g0573200 (CCS) interact with gene Os03g0219200 (OS03T0219200-01), Os06g0143000 (OS06T0143000-01), Os06g0115400 (OsJ_19901), Os05g0323900(SODA), Os03g0351500 (SODCC1), Os07g0665200 (SODCC2), OS08g0561700(SODCP). These genes are involved in the regulation of antioxidant enzymes. According to the above analysis, it is suggested that miR164d interacts with antioxidant enzyme genes through its target gene Os12g0610600 (OsJ_36832). (The above conclusions are obtained through online analysis of the website string, https://string-db.org/)

I have revised this sentence as follows:

Osa-miR164d, a miRNA which interact with the rice gene which encodes Cu-Zn superoxide dismutase, was down-regulated in 9311-4x (Table 1). MiR164d target four genes, including Os02g0579000, Os06g0675600, Os12g0610600, and Os04g0460600. The interaction analysis shows that gene Os12g0610600 (OsJ_36832) interact with gene Os04g0573200 (CCS), copper chaperone for superoxide dismutase, chloroplastic-like [Oryza sativa Japonica Group (Japanese rice)]. Further analysis shows that gene Os04g0573200 (CCS) interact with gene Os03g0219200 (OS03T0219200-01), Os06g0143000 (OS06T0143000-01), Os06g0115400 (OsJ_19901), Os05g0323900(SODA), Os03g0351500 (SODCC1), Os07g0665200 (SODCC2), OS08g0561700(SODCP). These genes are involved in the regulation of antioxidant enzymes. According to the above analysis, it is suggested that miR164d interacts with antioxidant enzyme genes through its target gene Os12g0610600 (OsJ_36832).(lines 320-329)

Fig1. Interaction analysis of miR164d target gene and genes regulating antioxidant enzymes

I have checked and confirmed that the fold change (FC) value in table 1 should be log2FC.(line 332-332)

Line 326, there is not Supplement Table S3.

Answer: Thank you for your good question. I have added Supplement Table S3. The table s3 list 38 target genes for 1864 miRNAs in Degradome result. （Line 349）
Lines 329-332 and 356-358, I did not find the data in Fig 5 or any other place to support the claims here. I suggest that the authors present the degradome data in a supplemental data set.

Answer: Thanks for your good suggestions. I have added Supplement Table S3. The Kyoto Encyclopedia of Genes and Genomes (KEGG) enrichment analysis was based on data from Table 3.（Line 359）

Lines 394-396, the authors should be aware that the expression of OsNCED3 and OxABA80x2 is far from ABA biosynthesis or degradation. I suggest that the authors revision the claims here. Besides, what is OxABA80x2? Please give the gene ID.

Answer: Thank you for your good question. I have further consulted three relevant papers, indicating that the expression of genes OsNCED3 and OxABA80x2 are related to the biosynthesis or degradation of endogenous ABA. (Nambara E, Marion-Poll A. Abscisic acid biosynthesis and catabolism[J]. Annual review of plant biology, 2005) Therefore, the expression of these two genes may be related to the deep dormancy of rice seed 9311-4x, I have revised this sentence as follows:

ABA induces seed dormancy and inhibits seed germination. The expression of gene OsNCED3 was consistent with the change trend of endogenous ABA content. The gene OsABA8ox2 is invovled in ABA catabolic pathways. The relative expression of genes OsNCED3 and OxABA80x2（Fig 8) were checked, with the result showing that the 9311-4x rice seeds possessed higher abscisic acid (ABA) synthesis potential and weaker ABA degradation potential than did 9311-2x. As a result, 9311-4 may be in deep dormancy state.(lines 417-422)

Fig2. ABA biosynthetic pathway. Synthesis of violaxanthin is catalyzed by zeaxanthin epoxidase (ZEP). A reverse reaction occurs in chloroplasts in high light conditions catalysed by violaxanthin de-epoxidase (VDE). The formation of cis-isomers of violaxanthin and neoxanthin may require two enzymes, a neoxanthin synthase (NSY) and an isomerase. Cleavage of cis-xanthophylls is catalysed by a family of 9-cis-epoxycarotenoid dioxygenases (NCED). Xanthoxin is then converted by a short-chain alcohol dehydrogenase (ABA2) into abscisic aldehyde, which is oxidized into ABA by an abscisic aldehyde oxidase (AAO3). AAO3 protein contains a molydenum cofactor activated by a MoCo sulfurase. A list of defective mutants, which have been named separately depending on pecies or selective screens, is given on the right side of each enzymatic step.

Figure 3. ABA catabolic pathways. Three different hydroxylation pathways are shown. The 8 -hydroxylation is thought to be the predominant pathway for ABA catabolism. Asterisks indicate targets for the conjugation. Red and blue asterisks indicate active and less-active hydroxy groups for conjugation, respectively.

Figure 2 and 3 cite from: Nambara E, Marion-Poll A. Abscisic acid biosynthesis and catabolism[J]. Annual review of plant biology, 2005

I am so sorry there is a spelling error, OxABA80x2 should be OsABA8ox2 (Os08g0472800) , I have revised it.(lines 418-419)

Lines 448-456, superoxide dismutase genes are not the target of miR164, instead, they are the target of miR398. The target of miR164 are members of NAC transcription factor gene family.

Answer: Thank you for your good question. A previous study revealed that the target of miR164 is members of NAC transcription factor gene family. Superoxide dismutase genes are the target of miR398. The interaction analysis in this paper shows that miR164d targeted NAC gene interact with gene antioxidant enzyme gene. I have revised this sentence as follows:

A previous study had revealed that the target of miR164 are members of NAC transcription factor gene family. miR164 was involved in strawberry fruit senescence by negatively mediating the expression of NAC transcription factors[52]. miR164-targeted NAC genes may be negative regulators of drought tolerance in rice[53]. Activation of the stress responses and antioxidant system through down-regulating the expression of miR164, miR6260, miR5929, miR6214, miR3946 and miR3446[54]. In this paper, miR164d targeted NAC gene interacts with antioxidant enzyme genes, including copper chaperone for superoxide dismutase. It is suggested that miR164d may negatively regulate antioxidant enzyme genes, thus delaying the senescence of rice seeds 9311-4x. (lines 473-481)

Finally, we would like to thank you and the reviewers again and we are looking forward to hearing your suggestion.

Best wishes,

Sincerely,

Yuchi He, Changfeng Zhang

E-mail: hyc@hubu.edu.cn, zcf202@163.com
